# Polyketides from the Mangrove-Derived Endophytic Fungus *Nectria* sp. HN001 and Their α-Glucosidase Inhibitory Activity

**DOI:** 10.3390/md14050086

**Published:** 2016-04-28

**Authors:** Hui Cui, Yayue Liu, Yang Nie, Zhaoming Liu, Senhua Chen, Zhengrui Zhang, Yongjun Lu, Lei He, Xishan Huang, Zhigang She

**Affiliations:** 1 School of Chemistry and Chemical Engineering, Sun Yat-Sen University, Guangzhou 510275, China; cuihui2@mail2.sysu.edu.cn (H.C.); liuyayue@mail2.sysu.edu.cn (Y.L.); drugs999@163.com (Y.N.); liuzhaom@mail2.sysu.edu.cn (Z.L.); chensh65@mail2.sysu.edu.cn (S.C.); zhangzhr7@mail2.sysu.edu.cn (Z.Z.); 2 School of Life Sciences and Biomedical Center, Sun Yat-Sen University, Guangzhou 510275, China; luyj@mail.sysu.edu.cn (Y.L.); helei8688@126.com (L.H.); 3 Guangdong Food and Drug Vocational College, Guangzhou 510275, China

**Keywords:** polyketides, α-glucosidase inhibitor, *Nectria* sp., pentaene diacid derivatives

## Abstract

Four new polyketides: nectriacids A–C (**1**–**3**) and 12-epicitreoisocoumarinol (**4**), together with three known compounds: citreoisocoumarinol (**5**), citreoisocoumarin (**6**), and macrocarpon C (**7**) were isolated from the culture of the endophytic fungus *Nectria* sp. HN001, which was isolated from a fresh branch of the mangrove plant *Sonneratia* *ovata* collected from the South China Sea. Their structures were determined by the detailed analysis of NMR and mass spectroscopic data. The absolute configuration of the stereogenic carbons for compound **4** was further assigned by Mosher’s ester method. All of the isolated compounds were tested for their α-glucosidase inhibitory activity by UV absorbance at 405 nm, and new compounds **2** and **3** exhibited potent inhibitory activity with IC_50_ values of 23.5 and 42.3 μM, respectively, which were more potent than positive control (acarbose, IC_50_, 815.3 μM).

## 1. Introduction

Diabetes mellitus, one of the most common chronic metabolic diseases, occurs when the pancreas produces insufficient levels of insulin or when the body cannot use the insulin effectively [1]. In 2015, about 415 million people had diabetes worldwide, with type II diabetes accounting for about 90% of the cases [2,3]. α-Glucosidase is an important enzyme for breaking down complex carbohydrates for absorption, and α-glucosidase inhibitors such as acarbose, miglitol, and voglibose, all originating from natural products, are widely used to treat type II diabetes, indicating that natural products are an important source of anti-diabetes drugs.

**Figure 1:**
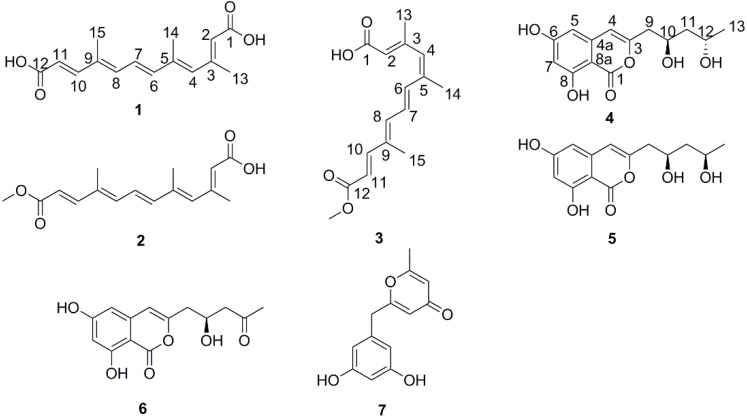
Chemical constituents of *Nectria* sp. HN001.

Endophytic fungi can produce a diversity of natural products, which are structurally unique and possess interesting biological and pharmacological properties [4,5]. As part of our ongoing investigation into bioactive metabolites from mangrove endophytic fungi collected from the South China Sea [6,7,8,9,10,11,12], a chemical investigation of the mangrove-derived fungus *Nectria* sp. HN001, isolated from a fresh branch of the mangrove plant *Sonneratia* *ovata*, had led to the isolation and characterization of four new polyketides: nectriacid A (**1**), nectriacid B (**2**), nectriacid C (**3**), and 12-epicitreoisocoumarinol (**4**), as well as three known compounds: citreoisocoumarinol (**5**), citreoisocoumarin (**6**), and macrocarpon C (**7**) (Figure 1). Previous studies showed that linear polyene derivatives exhibited anti-inflammatory [13], antihypertensive [14], antibacterial [15], and antifungal activities [16]. In this report, compounds (**1**–**7**) from the *Nectria* sp. HN001 were evaluated for α-glucosidase inhibitory activity. The results showed that compounds **2** and **3** exhibited significant inhibitory activity toward α-glucosidase. Here, details of the isolation, structure elucidation, and activity against α-glucosidase of these compounds are described.

## 2. Results

**Figure 2:**
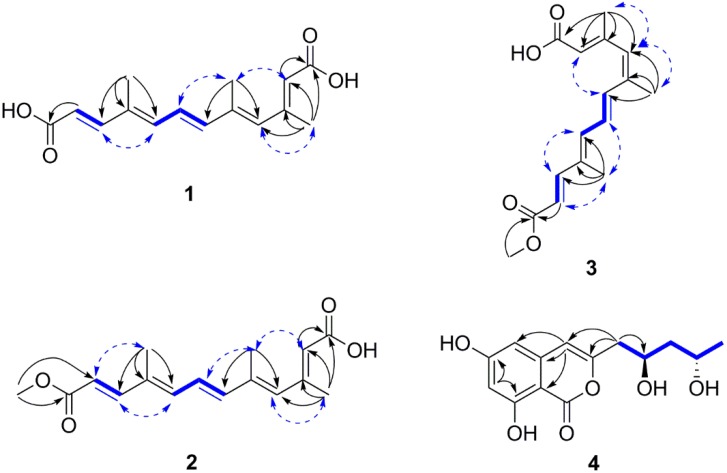
Selected ^1^H–^1^H COSY (bold line), HMBC (arrow), and key NOESY (dashed lines) correlations of compounds **1**–**4**.

Nectriacid A (**1**) was obtained as yellow amorphous powder. Its molecular formula C_15_H_18_O_4_ was established by the (−)-HRESIMS at *m*/*z* 261.1130 [M − H]^−^ (calcd for 261.1132), implying seven degrees of unsaturation. Its IR spectrum exhibited absorption bands for hydroxyl (3363 cm^−1^) and conjugated carbonyl (1684 cm^−1^) groups. The ^1^H NMR data of **1** (Table 1) showed resonances for three methyl groups [δ_H_ 1.93 (3H, s, H-15); δ_H_ 2.02 (3H, s, H-14); δ_H_ 2.24 (3H, s, H-13)], three olefinic protons [δ_H_ 6.57 (1H, d, *J* = 11.2 Hz, H-8), δ_H_ 6.20 (1H, s, H-4), and δ_H_ 5.76 (1H, s, H-2)], two *E*-configured olefinic protons [δ_H_ 7.27 (1H, d, *J* = 15.6 Hz, H-10) and δ_H_ 5.86 (1H, d, *J* = 15.6 Hz, H-11)], and another two *E*-configured olefinic protons [δ_H_ 6.76 (1H, dd, *J* = 15.1, 11.2 Hz, H-7) and δ_H_ 6.60 (1H, d, *J* = 15.1 Hz, H-6)]. The ^13^C NMR (Table 1) and DEPT data showed 15 carbon resonances corresponding to three methyl (δ_C_ 18.5, 14.2, 12.5), seven methine sp^2^ (δ_C_ 148.1, 141.8, 138.4, 136.1, 126.2, 120.2, 117.9), three quaternary sp^2^ (δ_C_ 151.6, 138.5, 134.4), and two carbonyls (δ_C_ 167.7, 167.4) carbons. The ^1^H and ^13^C NMR data of **1** were similar to those of all-*E*-4, 9-dimethyldodeca-2, 4, 6, 8, 10-pentaenedioic acid [17], which was isolated from the root of *Mycorrhizal* *colonization* except for the presence of an additional methyl group on C-5 (CH_3_-14) in **1**. The key HMBC correlations from H_3_-14 to C-6 and C-4 demonstrated that CH_3_-14 (δ_H_ 2.02, s, δ_C_ 14.2) was connected to C-5. Besides, comparing **1** with the known all-*E*-4, 9-dimethyldodeca-2, 4, 6, 8, 10-pentaenedioic acid, CH_3_-13 (δ_H_ 2.24, s, δ_C_ 18.5) of **1** was linked to C-3 rather than C-4, and this was supported by the HMBC correlations (Figure 2) from H_3_-13 to C-4, C-3, C-2, and C-1. Furthermore, the configurations of the double bonds (*E* or *Z*) for compound **1** were determined by the coupling constant and NOESY data. The *E*-geometry of the double bonds at C-6 and C-10 was assigned by the vicinal coupling constants, *J* = 15.6 Hz, *J* = 15.1 Hz, respectively. In addition, the geometry of the remaining three substituted double bonds was confirmed as 2*E*, 4*E* and 8*E* on the basis of the NOESY correlations (Figure 2) from H_3_-13 to H-4, H_3_-14 to H-2 and H-7, H-8 to H-10, as was previously reported [18]. Thus, compound **1** was determined as 2*E*, 4*E*, 6*E*, 8*E*, 10*E*-3, 5, 9-trimethyldodeca-2, 4, 6, 8, 10-pentaenedioic acid, and named nectriacid A.

Nectriacid B (**2**) was also isolated as yellow powder, and had a molecular formula of C_16_H_20_O_4_ according to its (−)-HRESIMS *m*/*z* 275.1287 [M − H]^−^. The ^1^H NMR data exhibited the signals for three methyl groups [δ_H_ 1.93 (3H, s, H-15); δ_H_ 2.04 (3H, s, H-14); δ_H_ 2.30 (3H, s, H-13)], one methoxy group (δ_H_ 3.74, s), three olefinic protons [δ_H_ 5.78 (1H, s, H-2); δ_H_ 6.08 (1H, s, H-4); δ_H_ 6.43 (1H, d, *J* = 11.2 Hz, H-8)], and two pairs of *E*-configured protons [δ_H_ 6.44 (1H, d, *J* = 15.2 Hz, H-6), δ_H_ 6.70 (1H, dd, *J* = 15.2, 11.2 Hz, H-7); δ_H_ 7.38 (1H, d, *J* = 15.6 Hz, H-10), δ_H_ 5.92 (1H, d, *J* = 15.6 Hz, H-11)]. The ^13^C NMR (Table 1) and HSQC spectra exhibited three methyl (δ_C_ 20.3, 14.8, 12.8), one methoxy (δ_C_ 51.8), seven methine sp^2^ (δ_C_ 149.7, 141.8, 138.8, 136.3, 126.6, 118.8, 116.6), five quaternary sp^2^ (δ_C_ 171.6, 168.0, 155.8, 135.1, 134.6) carbons. The above spectral features suggested that **2** was quite similar to **1** except for the presence of one methoxy group (δ_H_ 3.74, δ_C_ 51.8). This evidence suggested that compound **2** was derived from a methyl esterification of compound **1**, which was further supported by the HMBC correlations (Figure 2) from the methoxy protons to C-12 (δ_C_ 168.0). The 6*E* and 10*E* configurations were confirmed by the vicinal coupling constants between H-10 and H-11 (*J* = 15.6 Hz), H-6 and H-7 (*J* = 15.2 Hz). The configurations of 2*E*, 4*E* and 8*E* were assigned on the basis of the NOESY correlations (Figure 2) from H_3_-13 to H-4, H_3_-14 to H-2 and H-7, H-8 to H-10, H_3_-15 to H-11, as was previously reported [18]. Thus, the structure of **2** was established as 2*E*, 4*E*, 6*E*, 8*E*, 10*E*-12-methoxy-3, 5, 9-trimethyl-12-oxododeca-2, 4, 6, 8, 10-pentaenoic acid, and named nectriacid B.

Nectriacid C (**3**) was isolated as pale yellow powder, and its molecular formula of C_16_H_20_O_4_ was established by the (−)-HRESIMS *m*/*z* 275.1287 [M − H]^−^. The ^13^C NMR data (Table 1) of compound **3** showed 16 carbon resonances, which were classified according to DEPTs and HSQC spectra, as three methyl (δ_C_ 21.4, 20.1, 12.9), five quaternary sp^2^ (δ_C_ 170.9, 167.9, 155.7, 137.7, 135.1), seven methine sp^2^ (δ_C_ 149.0, 139.0, 134.6, 134.3, 118.9, 117.1), and one methoxy (δ_C_ 51.7) carbons. Comparison of NMR data with those of **2** suggested that compound **3** possessed the same planar structure as **2**. However, the double bonds geometry was different. The 6*E* and 10*E* configurations were confirmed by the vicinal coupling constants between H-6 and H-7 (*J* = 15.1 Hz), H-10 and H-11 (*J* = 15.6 Hz), respectively. The 2*E*, 4*Z*, and 8*E* configurations of the double bonds were established by the NOESY correlations from H_3_-13 and H_3_-14 to H-4, from H-2 to H-6, from H_3_-15 to H-7 and H-11, from H-10 to H-8 (Figure 2) [18]. Consequently, **3** was determined as 2*E*, 4*Z*, 6*E*, 8*E*, 10*E*-12-methoxy-3, 5, 9-trimethyl-12-oxododeca-2, 4, 6, 8, 10-pentaenoic acid, and named nectriacid C.

**Figure 3:**
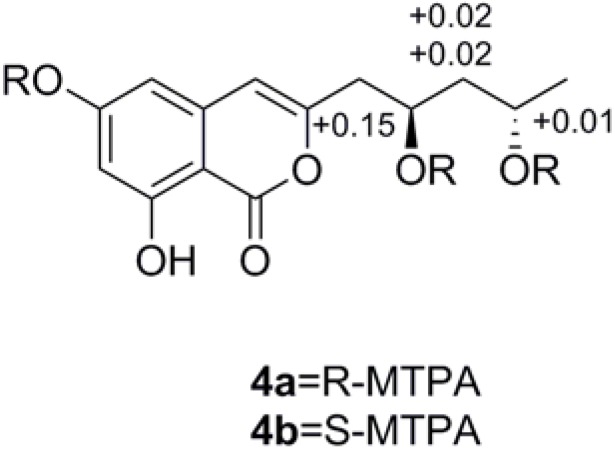
Δδ_S-R_ values of (*R*)- and (*S*)-MTPA esters of **4**.

Compound **4** was obtained as colorless powder. Its molecular formula C_14_H_16_O_6_ was deduced by the (−)-HRESIMS *m*/*z* 279.0871 [M − H]^−^, corresponding to seven degrees of unsaturation. The IR spectrum displayed typical absorption bands for hydroxyl (3394 cm^−1^) and aromatic ring system (1684, 1629, and 1588 cm^−1^). The ^1^H NMR spectrum (Table 2) suggested the presence of one methyl group [δ_H_ 1.21 (3H, d, *J* = 6.3 Hz, H-13)], two methylene groups [δ_H_ 2.65 (1H, dd, *J* = 14.5, 4.9 Hz, H-9a), 2.59 (1H, dd, *J* = 14.5, 8.0 Hz, H-9b); δ_H_ 1.60 (1H, ddd, *J* = 14.3, 10.6, 3.4 Hz, H-11a), 1.55 (1H, ddd, *J* = 14.3, 10.6, 3.4 Hz, H-11b], two methine groups [δ_H_ 4.21 (1H, m, H-10), 4.02 (1H, m, H-12)], and three olefinic protons [δ_H_ 6.37 (1H, s, H-4), 6.31 (2H, overlap, H-5, H-7)]. The ^13^C NMR spectrum of **4** (Table 2) showed 14 carbon resonances, including one methyl (δ_C_ 24.5), six quaternary sp^2^ (δ_C_ 168.0, 167.5, 165.0, 156.3, 141.4, 100.0), two methylene sp^3^ (δ_C_ 47.1, 43.1), three methine sp^2^ (δ_C_ 107.2, 103.8, 102.8), and two oxygen-bearing methine sp^3^ (δ_C_ 67.2, 65.4) carbons. Detailed analysis of the ^1^H and ^13^C NMR data (Table 2) suggested that **4** belonged to the isocoumarin class, and its NMR data were similar to those of citreoisocoumarinol **5** [19]. Comparison of the NMR data of compounds **4** and **5** suggested that they differed only in the substituents on C-3. The absolute configuration of C-10 and C-12 of compound **4** was determined as 10*R*, 12*S* by applying Mosher’s methods to the 1, 3-anti-diol model as reported [20,21,22]. The procedure started with esterification of **4** with the two enantiomers of (*R*)- or (*S*)-MTPA chloride (**4a** = *R* or **4b** = *S*) [23], and then the differences in chemical shift values (Δδ *=* δ_S_ − δ_R_) for **4b** and **4a** were calculated to assign the configuration of C-10 and C-12 (Figure 3). So **4** was named 12-epicitreoisocoumarinol. 

The genus *Nectria* has been reported as a prolific source of bioactive secondary metabolites, such as heptaketides with antimicrobial activity [24], terpenoids with anti-acetylcholinesterase and anti-β-glucuronidase activity [25], and phytotoxin with herbicidal activity [26]. Nectriacids A–C (**1**–**3**) were the first examples of polyenes derivatives with linear C_15_ conjugated pentaene diacid derivatives containing three methyl groups. To date only a few examples of similar substances have been previously isolated. These include bixin and norbixin from *Bixa* *orellana* [27], crocetin from *Crocus* *sativus* [28], the monocarboxylic acid azafrin from *Escobedia* *scabrifolia* and *E.* *linearis* [29], and (3*Z*, 5*E*, 7*E*, 9*E*, 11*E*, 13*Z*, 15*E*, 17*E*)-18-methyl-19-oxoicosa-3, 5, 7, 9, 11, 13, 15, 17-octaenoic acid and (3*E*, 5*Z*, 7*E*, 9*E*, 11*E*, 13*E*, 15*Z*, 17*E*, 19*E*)-20-methyl-21-oxodocosa-3, 5, 7, 9, 11, 13, 15, 17, 19-nonaenoic acid from white-rotting *Basidiomycete* [30].

The remaining three known compounds from the fungus *Nectria* sp. HN001 were identified as citreoisocoumarinol (**5**) [19,31], citreoisocoumarin (**6**) [19,31], and macrocarpon C (**7**) [19,31], by comparison of their MS and NMR data with those reported in the literature.

All of the isolates were evaluated for *in* *vitro* α-glucosidase inhibitory activity [10]. The results showed that compounds **2** and **3** possessed stronger activity than positive control (acarbose, IC_50_, 815.3 μM) with IC_50_ values of 23.5 and 42.3 μM, respectively. However, compounds **4**–**6** showed moderate activity with IC_50_ values ranging from 300 to 600 μM (Table 3), while compound **7** did not display inhibitory activity compared to positive control. Interestingly, although compounds **1**–**3** possess same carbon skeleton, their α-glucosidase inhibitory activity are different. The activity against α-glucosidase for **2** (23.5 μM) and **3** (42.3 μM) was more potent than that for **1** (121.8 μM), which suggested that esterification of terminal carboxyl group (C-12) may play a key role in the inhibitory effects. Although compounds **2** and **3** possess different configuration of the C4–C5 double bonds, they exhibited the same level of activity. Meanwhile, compounds **4** and **5** exhibited relatively stronger activity when compared to **6**.

## 3. Experimental Section

### 3.1. General

Optical rotations were measured on a Bellingham-Stanley ADP 440+ polarimeter at 25 °C. IR data were recorded on a Nicolet 5DX-FTIR (Thermo Fisher Scientific, Inc., Hudson, NH, USA), in KBr discs. UV data were recorded on a Shimadzu UV-240 spectrophotometer (Shimadzu, Kyoto, Japan). The ^1^H NMR (500 MHz), ^13^C NMR (125 MHz), and 2D NMR spectra were obtained on a Bruker AVANCE-500 (Bruker BioSpin Corporation, Billerica, MA, USA) using TMS as an internal reference. HRESIMS were acquired on a Thermofisher LTQ Orbitrp Elite LC-MS spectrometer (Thermo Fisher Scientific, Inc., Hudson, NH, USA), and the ESIMS data were measured on a Micro Mass Q-TOF spectrometer (Waters Corporation, Milford, MA, USA). TLC analysis was carried out on silica gel plates (Marine Chemical Ltd., Qingdao, China). RP-C18 silica gel (Fuji, 40–75 μm, Fuji Silysia Chemical Ltd., Kasugai, Japan), Silica gel (200–300 mesh, Marine Chemical Ltd., Qingdao, China), High silica gel (H, Marine Chemical Ltd., Qingdao, China), and Sephadex LH-20 (GE Healthcare Bio-Sciences AB, Stockholm, Sweden) were used for column chromatography (CC). The chiral HPLC separation of compound **4** was accomplished over a S-Chiral A (column size: 4.6 × 250 mm 5 μm; Acchrom Technologies Co., Ltd., Beijing, China; flow rate: 1.0 mL/min; solvent: *n*-hexane-isopropanol = 9:1, *t*_R_ 18.5 min). α-Glucosidase from *Saccharomyces* *cerevisiae* was purchased from Sigma-Aldrich Co. (CAS number: 9001-42-7, E.C 3.2.1.20; Buchs, Switzerland). Acarbose (>98%) was purchased from Adamas-beta Co. Ltd. (Shanghai, China).

### 3.2. Fungal Material

The fungal strain HN001 was isolated from the branches of the mangrove plant *Sonneratia* *ovata* collected from the South China Sea in Hainan province, China. The fungus was identified by our team as *Nectria* sp. HN001, Nectriaceae, according to a molecular biological protocol by DNA amplification and sequencing of the ITS region [10] (deposited in GenBank, accession No. KU359411). A voucher strain was deposited in School of Chemistry and Chemical Engineering, Sun Yat-Sen University, Guangzhou, China, with the access code, 2015-HN001.

### 3.3. Fermentation, Extraction, and Isolation

The fungus *Nectria* sp. HN001 was primary cultivated on PDA medium (20 g of glucose, 20 g of agar, and 2 g of sea salt in 1 L of potato infusion). Plugs of agar supporting mycelial growth were cut and transferred aseptically to 250 mL Erlenmeyer flasks containing 100 mL of PDB medium (20 g of glucose and 2 g of sea salt in 1 L of potato infusion). The flasks were incubated at 28 °C on a rotary shaker for three days, and then the mycelia were aseptically transferred to a solid autoclaved rice substrate medium (60 × 500 mL Erlenmeyer flasks, each containing 50 g of rice and 50 mL of 0.3% of saline water) for 28 days at 25 °C. The mycelia and solid rice medium were extracted with MeOH (3 × 15 L, 24 h each) for three times. The solvent was evaporated under reduced pressure to yield a crude extract (296 g), which was suspended in H_2_O and extracted with EtOAc (3 × 3 L, 45 min each) to yield a crude ethyl acetate extract (110 g). The ethyl acetate extract (110 g) was chromatographed on silica gel column (400 g, 100–200 mesh, 10 × 70 cm) eluting with a step gradient of petroleum ether-EtOAc (100:0; 9:1; 8:2; 7:3; 6:4; 5:5; 4:6; 3:7; 0:100, *v*/*v*, each 2 L) to give nine fractions (F1–F9). F8 (1.5 g) was further fractionated on another silica gel column using petroleum-EtOAc (2 L, 8:2; 7:3; 6:4; 5:5; 4:6, *v*/*v*, each 400 mL) as the mobile phase to yield five subfractions (F801 to F805). Compound **1** (28 mg) was obtained from F802 by column chromatography on silica gel (gel H, 25 × 340 mm, 30 g) eluting with CHCl_3_-MeOH (1 L, 95:5). Then, the F803 fraction was purified using HPLC on a semipreparative RP-HPLC column (250 × 9.4 mm, 5 μm), using with Acetonitrile-H_2_O (55:45, *v*/*v*, flow rate: 1.0 mL/min) as the solvent system, to obtain **2** (18 mg, *t*_R_ 26.3 min) and **3** (15 mg, *t*_R_ 27.8 min). F804 was subjected to RP-18 using MeOH-H_2_O (8:2, *v*/*v*) to give compounds **6** (35 mg) and **7** (8 mg). F805 was chromatographed on Sephadex LH-20 (110 g, 110 × 3 cm) eluting with CHCl_3_-MeOH (1 L, 1:1, *v*/*v*) to give the mixture of F8051 (**4** and **5**). F8051 was conducted by the S-Chiral A (n-hexane-isopropanol = 9:1, *v*/*v*, flow rate: 1.0 mL/min) column to afford **4** (18 mg, *t*_R_ 18.5 min) and **5** (10 mg, *t*_R_ 25.5 min).

Nectriacid A (**1**): yellow powder; UV (MeOH) λ_max_ (log ε) 355 (4.70); IR (KBr) ν_max_ 3363, 2955, 2922, 2852, 1684, 1616, 1592, 1268, 1186, 980, 895, 855 cm^−1^; ^1^H NMR and ^13^C NMR data, see Table 1; ESIMS *m*/*z* 261.1 [M − H]^−^; HRESIMS *m/z* 261.1130 [M − H]^−^ (calcd for C_15_H_17_O_4_, 261.1132).

Nectriacid B (**2**): yellow powder; UV (MeOH) λ_max_ (log ε) 359 (4.23); IR (KBr) ν_max_ 3434, 2955, 2922, 2852, 1723, 1656, 1439, 1380, 1278, 1202, 1173, 984, 857 cm^−1^; ^1^H NMR and ^13^C NMR data, see Table 1; ESIMS *m*/*z* 275.3 [M − H]^−^; HRESIMS *m*/*z* 275.1287 [M − H]^−^ (calcd for C_16_H_19_O_4_, 275.1288).

Nectriacid C (**3**): pale yellow powder; UV (MeOH) λ_max_ (log ε) 355 (3.69); IR (KBr) ν_max_ 3365, 2956, 2922, 2852, 1723, 1655, 1634, 1464, 1439, 1380, 1280, 1202, 1174, 1073, 984, 720 cm^−1^; ^1^H NMR and ^13^C NMR data, see Table 1; ESIMS *m*/*z* 275.4 [M − H]^−^; HRESIMS *m*/*z* 275.1287 [M − H]^−^ (calcd for C_16_H_19_O_4_, 275.1288).

12-epicitreoisocoumarinol (**4**): colorless powder; ${\left[\alpha \right]}_{D}^{25}$ −13.3 (*c* 0.3, MeOH); UV (MeOH) λ_max_ (log ε) 245 (4.89); IR (KBr) ν_max_ 3394, 3191, 2962, 2921, 2850, 1681, 1629, 1588, 1510, 1466, 1382, 1241, 1172, 1134, 1065, 849, 798, 694 cm^−1^; ^1^H NMR and ^13^C NMR data, see Table 2; ESIMS *m*/*z* 279.4 [M − H]^−^; HRESIMS *m*/*z* 279.0871 [M − H]^−^ (calcd for C_14_H_15_O_6_, 279.0874).

### 3.4. Preparation of (R)- and (S)-MTPA Esters of **4**

As our previous reported method [23], 12-epicitreoisocoumarinol (**4**) (1 mg) was dissolved in 1 mL of pyridine and stirred at room temperature for 10 min. An excess of (*R*)- or (*S*)-MTPA chloride (10 μL) was added, and the reaction was stirred overnight at room temperature. The solvent was removed in vacuo, and the crude reaction product was purified by preparative silica gel TLC using pure dichloromethane as developing solvent.

^1^H NMR data of (*S*)-MTPA ester of **4** (500 MHz, CDCl_3_): δ_H_ 11.0 (1H, s, 8-OH), 7.60–7.29 (15H, m, Ar-H), 6.70 (1H, d, *J* = 2.0 Hz, H-5), 6.42 (1H, d, *J* = 2.0 Hz, H-7), 5.87 (1H, s, H-4), 5.34 (1H, m, H-10), 5.07 (1H, m, H-12), 3.66-3.45 (9H, s, 3-OCH_3_), 2.72 (1H, dd, *J* = 15.1, 5.0 Hz, H-9a), 2.71 (1H, dd, *J* = 15.1, 6.7 Hz, H-9b), 1.99 (1H, m, H-11a), 1.96 (1H, m, H-11b), 1.27 (3H, d, *J* = 6.2 Hz, H-13); ESIMS *m/z* 927.2 [M − H]^−^.

^1^H NMR data of (*R*)-MTPA ester of **4** (500 MHz, CDCl_3_): δ_H_ 11.0 (1H, s, 8-OH), 7.61–7.29 (15H, m, Ar-H), 6.71 (1H, d, *J* = 2.1 Hz, H-5), 6.48 (1H, d, *J* = 2.1 Hz, H-7), 5.97 (1H, s, H-4), 5.18 (1H, m, H-10), 5.06 (1H, m, H-12), 3.66-3.48 (9H, s, 3-OCH_3_), 2.71 (1H, overlap, H-9a), 2.70 (1H, overlap, H-9b), 1.96 (1H, m, H-11a), 1.93 (1H, m, H-11b), 1.32 (3H, d, *J* = 6.2 Hz, H-13); ESIMS *m*/*z* 927.2 [M − H]^−^.

### 3.5. In Vitro Inhibition Studies on α-Glucosidase

An assay of α-glucosidase inhibitory activity was performed using a reported method, with slight modifications [10]. All the assays were performed using 0.01 M KH_2_PO_4_-K_2_HPO_4_ buffers, pH 7.0, and a Bio-Rad iMark microplate reader (Bio-Rad Laboratories, Inc., Kyoto, Japan). Enzyme solution was prepared to give 2.0 Units/mL in 2 mL aliquots. The assay medium contained phosphate buffer, pH 7.0 (130 μL), 10 μL of enzyme solution, 20 μL of DMSO or inhibitor (dissolved in DMSO), and 40 μL of substrate (*p*-nitrophenyl glycoside, 3 mg/mL). The substrate was added to the assay medium containing enzyme and buffer with inhibitor added after 15 min of incubation time at 37 °C. The activity was determined by measuring the increase in absorbance at 405 nm for a 1 min interval. Calculations were performed according to the equation:

η (%) = [(B − S)/B] × 100%

(*B* stands for the assay medium with DMSO; *S* stands for the assay medium with inhibitor). All measurements were done in triplicate from two independent experiments. The reported IC_50_ was the average value of two independent experiments.

## 4. Conclusions 

Four new (**1**–**4**) and three known polyketides (**5**–**7**) were isolated and identified from the culture of the endophytic fungus *Nectria* sp. HN001. Compounds **2** and **3** exhibited stronger inhibitory activity on α-glucosidase than positive control [10]. To the best of our knowledge, this is the first report of the α-glucosidase inhibitory activity of the C_15_ conjugated pentaene diacid derivatives. This finding can allow us to explore structural diversity of linear polyene diacid derivatives and offer new guidance to discover α-glucosidase inhibitors.

## Figures and Tables

**Table 1:** ^1^H (500 MHz) and ^13^C (125 MHz) NMR data for compounds **1** (DMSO-*d*_6_), **2** (CDCl_3_), and **3** (CDCl_3_).

Position	1		2		3	
	δ_H_ (*J* in Hz)	δ_C_	δ_H_ (*J* in Hz)	δ_C_	δ_H_ (*J* in Hz)	δ_C_
1		167.7, C		171.6, C		170.9, C
2	5.76, s	120.2, CH	5.78, s	118.8, CH	5.76, s	118.9, CH
3		151.6, C		155.8, C		155.7, C
4	6.20, s	136.1, CH	6.08, s	136.3, CH	5.97, s	134.3, CH
5		138.5, C		134.6, C		137.7, C
6	6.60, d (15.1)	141.8, CH	6.44, d (15.2)	141.8, CH	6.91, d (15.1)	134.6, CH
7	6.76, dd (15.1, 11.2)	126.2, CH	6.70, dd (15.2, 11.2)	126.6, CH	6.73, dd (15.1,11.4)	127.8, CH
8	6.57, d (11.2)	138.4, CH	6.43, d (11.2)	138.8, CH	6.49, d (11.4)	139.0, CH
9		134.4, C		135.1, C		135.1, C
10	7.27, d (15.6)	148.1, CH	7.38, d (15.6)	149.7, CH	7.38, d (15.6)	149.0, CH
11	5.86, d (15.6)	117.9, CH	5.92, d (15.6)	116.6, CH	5.92, d (15.6)	117.1, CH
12		167.4, C		168.0, C		167.9, C
13	2.24, s	18.5, CH_3_	2.30, s	20.3, CH_3_	2.26, s	20.1, CH_3_
14	2.02, s	14.2, CH_3_	2.04, s	14.8, CH_3_	2.00, s	21.4, CH_3_
15	1.93, s	12.5, CH_3_	1.93, s	12.8, CH_3_	1.93, s	12.9, CH_3_
-OCH_3_			3.74, s	51.8, CH_3_	3.74, s	51.7, CH_3_

**Table 2:** ^1^H (500 MHz) and ^13^C (125 MHz) NMR data for compound **4** (MeOD-*d*_4_) and compound **5** (MeOD-*d*_4_).

	4		5	
Position	δ_H_ (*J* in Hz)	δ_C_	δ_H_ (*J* in Hz)	δ_C_
1		168.0, C		168.0, C
3		156.3, C		156.1, C
4	6.37, s	107.2, CH	6.37, s	107.3, CH
4a		141.4, C		141.4, C
5	6.31, s	103.8, CH	6.31, s	103.8, CH
6		167.5, C		167.5, C
7	6.31, s	102.8, CH	6.31, s	102.8, CH
8		165.0, C		165.0, C
8a		100.0, C		100.0, C
9	2.59, dd (14.5, 8.0)	43.1, CH_2_	2.57, dd (14.5, 8.3)	42.5, CH_2_
	2.65, dd (14.5, 4.9)		2.70, dd (14.5, 4.4)	
10	4.21, m	67.2, CH	4.14, m	68.6, CH
11	1.60, ddd (14.3, 10.6, 3.4)	47.1, CH_2_	1.71, ddd (13.9, 8.8, 7.6)	46.4, CH_2_
	1.55, ddd (14.3,10.6, 3.4)		1.61, ddd (13.9, 5.4, 4.3)	
12	4.02, m	65.4, CH	4.00, m	67.0, CH
13	1.21, d (6.3)	24.5, CH_3_	1.20, d (6.2)	23.5, CH_3_

**Table 3:** Inhibitory effects of the isolates against α-glucosidase.

Compounds	1	2	3	4	5	6	7	Acarbose ^a^
IC_50_ (μM) ^b^	121.8 ± 0.4	23.5 ± 0.3	42.3 ± 0.2	343.7 ± 1.0	392.5 ± 1.7	538.7 ± 4.3	>900	815.3 ± 3.8

^a^ Positive control; ^b^ Data are shown as mean ± SD from three parallel measurements.
